# Supplementary figures and images for: Pfs48/45 nanobodies block Plasmodium falciparum transmission
Source: PLoS Pathog. 2026 Jan 27;22(1):e1013884. doi: 10.1371/journal.ppat.1013884 (PMC12858062; doi:10.1371/journal.ppat.1013884)

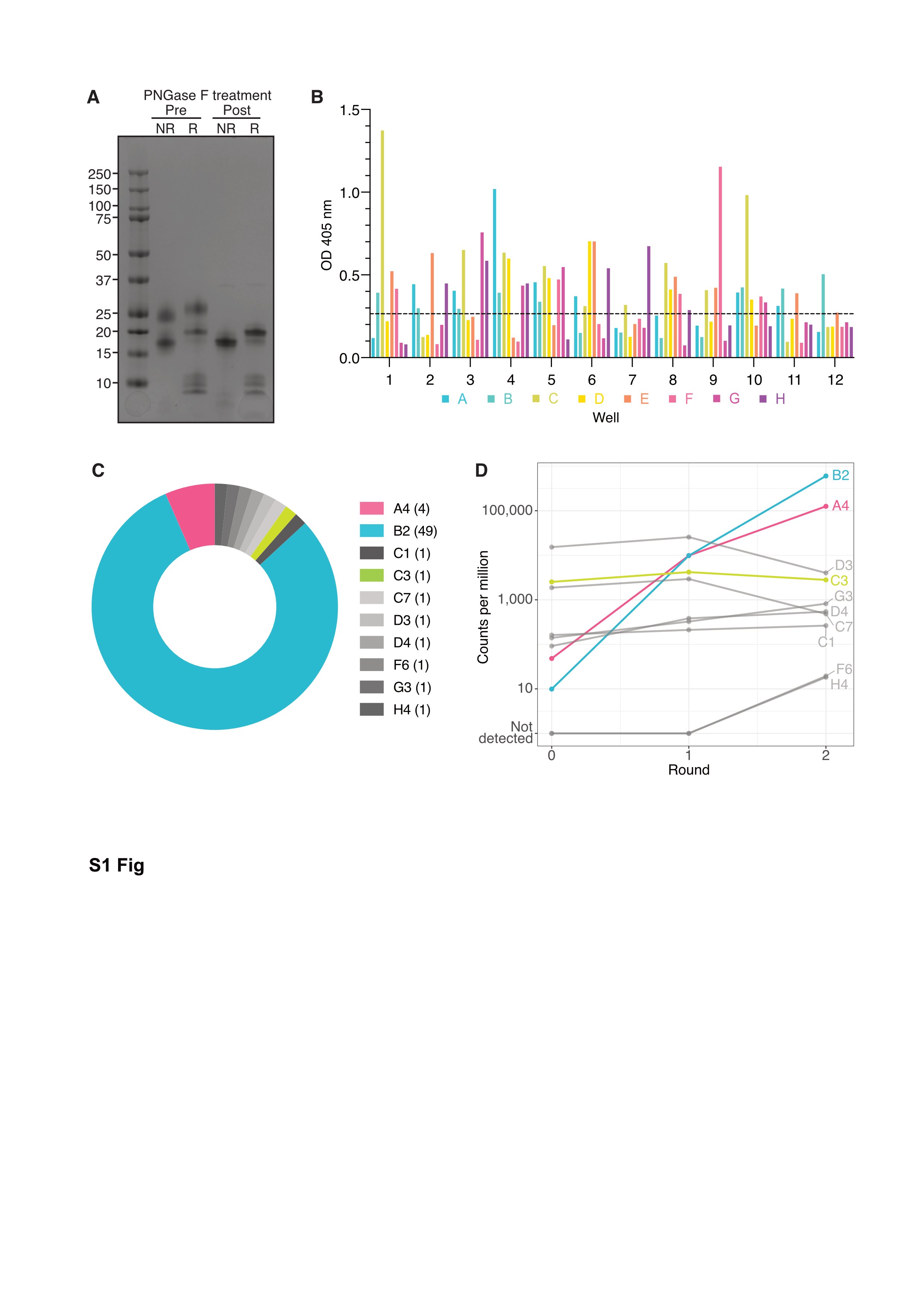

Supplement: S1 Fig — (A) Purified Pfs48/45 D3 pre and post PNGase F-deglycosylation under non-reducing (NR) and reducing (R) conditions. Pre-treatment, glycosylated forms of Pfs48/45 are visible as additional bands above the expected molecular weight of 19.4 kDa. After PNGase F treatment, a single band is observed at the expected molecular weight. (B) ELISA screen of 94 phage supernatants to identify clones that are positive for Pfs48/45 D3 binding. Dashed line denotes cut-off for positive hits, defined as double the mean OD of the PBS (H1) and irrelevant nanobody negative control (H12). (C) Overview of enrichment in the 10 anti-Pfs48/45 D3 clonal groups identified by Sanger sequencing. Transmission-blocking nanobodies are highlighted in colour. The number of clones per group are indicated in brackets. (D) Line graph of the normalised counts per million (CPM) from next-generation sequencing (NGS) of the 10 anti-Pfs48/45 nanobodies across round 1 and round 2 of phage display. (TIFF) [file ppat.1013884.s001.tiff]

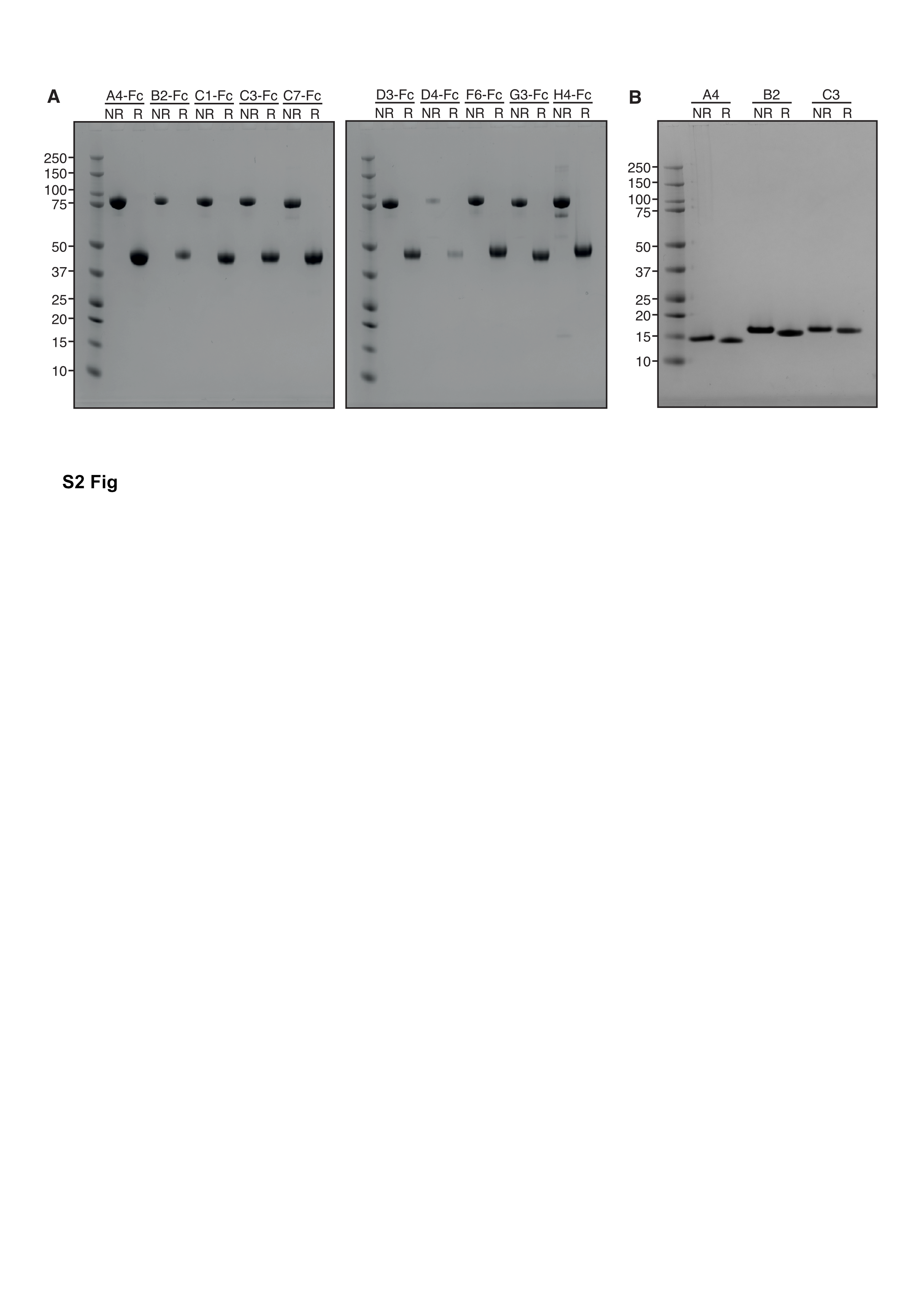

Supplement: S2 Fig — (A) Purified anti-Pfs48/45 nanobody-Fcs (~80 kDa) under non-reducing and reducing conditions. (B) Purified monomeric anti-Pfs48/45 nanobodies (~15 kDa) under non-reducing and reducing conditions. (TIFF) [file ppat.1013884.s002.tiff]

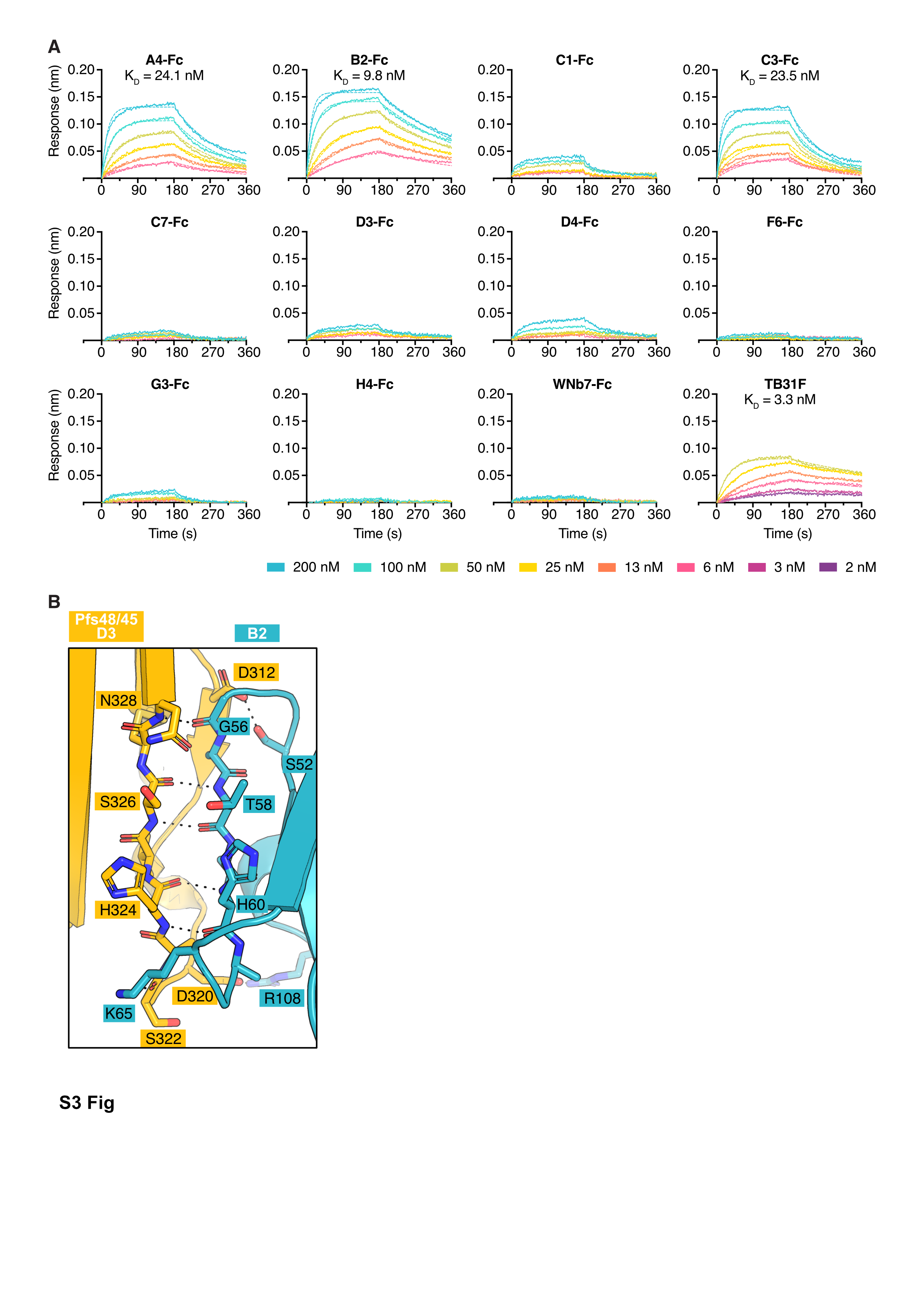

Supplement: S3 Fig — (A) Representative binding curves of different concentrations of Pfs48/45 D3 to immobilised nanobody-Fcs and TB31F. Binding curves were generated by bio-layer interferometry and curves were fitted using a 1:1 Langmuir binding model. Binding affinities (KD) are indicated above binding curves. (B) Close-up view of the crystal structure of the Pfs48/45- nanobody B2 complex showing hydrogen bonds and ionic interactions between them. Ribbon representation of Pfs48/45 D3 in yellow and nanobody B2 in blue. (TIFF) [file ppat.1013884.s003.tiff]
