# Supplementary material for: Pfs48/45 nanobodies block Plasmodium falciparum transmission
Source: PLoS Pathog. 2026 Jan 27;22(1):e1013884. doi: 10.1371/journal.ppat.1013884 (PMC12858062; doi:10.1371/journal.ppat.1013884)
Supplement: S1 Table — (DOCX) [file ppat.1013884.s004.docx]

Table S1. Anti-Pfs48/45 nanobody (Nb) amino acid sequences

| **Nb** | **Full Nb sequence** |
| --- | --- |
| A4 | QVQLQESGGGLVQPGGSLRLSCAASGFTFSSYWMYWVRETPGKGLEWVSVINPGGRITIYTDSVKGRFTVSRDNAKNTVYLQMNSLKPEDTAVYFCAADLEYRAQLYAYWGQGTQVTVSS |
| B2 | QVQLQESGGGLVQAGGSLRLSCAASGRTFSKYAMAWFRQAPGKEREFVAAISGAGGATVHAAPVKGRFTISRDNAKNMGYLQMNSLKPEDTAVYYCAQTGSTGWPAGRTYTYDYWGQGTQVTVSS |
| C1 | QVQLQESGGGLVQAGGSLRLSCAASGRTFSKYAMAWFRQAPGKEREFVAAISGAGGATVHAAPVKGRFTISRDNAKNMGYLQMNSLKPEDTAVYYCAASHAVVALKAYTNWGQGTQVTVSS |
| C3 | QVQLQESGGGSVQPGGSLRLSCAASGFTFSSYWMYWVRQAPEKGLEWVSVINPGGRITIYTDSVKGRFAVSRDNAKNTVYLQLNSLKPEDTAVYYCAASRTYSNSYSPSDYDYWGQGTQVTVSS |
| C7 | QVQLQESGGGLVQAGGSLRISCAGSGFSFDDYAIAWFRQAPGKEREVVSCISSDGTIYYEDSVKGRFVLSSDKAKSTLSLQMNSLKPEDTAVYYCAADILALHYCAGYTDYDHWGLGTQVTVSS |
| D3 | QVQLQESGGGLVQPGGSLRLSCAASGFTFHDSTIGWFRQAPGKEREGVGCISSVDDSTAYADSVKDRFTISMDNTKTTVYLQMNSLKPEDTAVYTCATSRGVSACRIARRDYGFWGQGTQVTVSS |
| D4 | QVQLQESGGGLVQVGGSLRLACVASGFTFNSYWMYWVRQAPGKELEWVSAINPSGVIILYKNSVKGRFTISRDNVKNILYLEMNNLKPEDTATYYCAPTSPGIYSLFPKPGRYENWGQGTQVTVSS |
| F6 | QVQLQESGGGLVQPGGSLRLSCAASGGTFSRYAMGWFRQGPGKDREFVAGISWSGISTFYKEHAKGRFTISRDNAKNEVYLDMNNLGPEDTAVYYCAADDWQFDPLSGLGEYDYWGQGTQVTVSS |
| G3 | QVQLQESGGGLVQPGGSLRLSCAASGFTFSSYWMYWVRETPGKGLEWVSVINPGGRITIYTDSVKGRFTVSRDNAKNTVYLQMNSLQPEDTAVYYCAADLGIATMSDVALNYRHWGQGTQVTVSS |
| H4 | QVQLQESGGGQVQPGGSLRLSCEVSGFTLDVYAIGWFRQAPGKERERVSCISSNDGTTYYAESVKGRFTISSDNAKNTVFLQMNSLKPEDTAVYTCASFYAANEPYGSSCSAGQTYDYWGQGTQVTVSS |
