# Supplementary material for: Pfs48/45 nanobodies block Plasmodium falciparum transmission
Source: PLoS Pathog. 2026 Jan 27;22(1):e1013884. doi: 10.1371/journal.ppat.1013884 (PMC12858062; doi:10.1371/journal.ppat.1013884)
Supplement: S2 Table — (DOCX) [file ppat.1013884.s005.docx]

Table S2. Summary of interactions between Pfs48/45 D3 and nanobody B2

| Pfs48/45 D3 | Group | B2 | Group | Distance (Å) |
| --- | --- | --- | --- | --- |
|  |  |  |  |  |
| *Hydrogen bonds* | | | | |
| Asp 312 | OD2 | Ser 52 | OG | 2.3 |
| Asp 312 | OD2 | Gly 55 | N | 3.5 |
| Asp 312 | OD2 | Gly 56 | N | 2.8 |
| Asp 312 | OD2 | Ala 57 | N | 3.5 |
| Ser 322 | O | Lys 65 | NZ | 3.0 |
| His 324 | O | His 60 | N | 2.8 |
| His 324 | N | His 60 | O | 2.6 |
| Ser 326 | O | Thr 58 | N | 2.9 |
| Ser 326 | O | Thr 58 | OG1 | 3.8 |
| Ser 326 | N | Thr 58 | O | 3.0 |
| Asn 328 | N | Gly 56 | O | 2.6 |
| *Salt bridges* | | | | |
| Asp 320 | OD1 | Arg 108 | NH2 | 3.9 |
| Other B2 interfacing residues (Pfs48/45 D3) | | | | |
| Cys 298 | Thr 311 | Ser 313 | Leu 314 | Asp 315 |
| Leu 318 | Val 319 | Asp 320 | Ala 323 | Ile 325 |
| Cys 327 |  |  |  |  |
| Other Pfs48/45 D3 interfacing residues (B2) | | | | |
| Phe 47 | Gly 53 | Ala 54 | Val 59 | Ala 61 |
| Ala 62 | Thr 102 | Gly 103 | Trp 104 | Pro 105 |
| Ala 106 | Arg 108 |  |  |  |
