# Supplementary material for: Pfs48/45 nanobodies block Plasmodium falciparum transmission
Source: PLoS Pathog. 2026 Jan 27;22(1):e1013884. doi: 10.1371/journal.ppat.1013884 (PMC12858062; doi:10.1371/journal.ppat.1013884)
Supplement: Attachment — Submitted filename: Reviewer #2 Wai-Hong Tham.pdf [file ppat.1013884.s006.pdf]

Recommendation: Major Revision

In this manuscript the authors have explored the feasibility of developing nanobodies against the sexual stage antigens, Pfs48/45 and Pfs230, to inhibit transmission of P. falciparum. Following immunization of alpacas with deglycosylated Pfs48/45 D3 a nanobody phage library was generated and screened for binding to Pfs48/45 by ELISA. Anti-Pfs48/45 nanobodies were produced as recombinant proteins fused to Fc and tested for recognition of Pfs48/45 by ELISA and immunofluorescence assay (IFA). The interaction of three nano-Fcs, A4-Fc, B2-Fc and C3-Fc, that bind Pfs48/45 D3 domain was analyzed. The three nanobodies react to overlapping epitopes on Pfs48/45 D3 but don't inhibit binding of a previously identified mAb referred to as TB31F. The three nanobodies, A4-Fc, B2-Fc and C3-Fc, like TB31F, block parasite transmission with high efficiency. The binding site of one of the nanobodies, B2-Fc, was determined by solving the structure of the nanobody in a complex with Pfs48/45 D3. By linking previously identified nanobodies, F5 and F10, against Pfs230, with B2 the authors produced chimeric bispecific nanobodies, that react with both Pfs48/45 and Pfs230. Importantly, the chimeras retain the ability to target the mosquito stages and block transmission efficiently. The authors have shown elegantly the feasibility of using nanobodies to inhibit P. falciparum transmission. They should address the following main comment:

The characterization of the nanobodies in terms of binding to the antigens and mapping of the binding epitopes is clearly described. However, the function studies testing the ability of the nanobodies to block parasite transmission need additional work. The nanobodies were tested for inhibition of transmission at a single dilution at which close to 100% inhibition is achieved. It would have been informative to test the nano-Fcs at different concentrations to identify the most potent nano-bodies for transmission blocking activity. The experiment with bispecific nano-Fcs was also performed at a single concentration. At the concentration used even mono-specific nanobodies yield close to 100% inhibition. It is therefore difficult to determine if there is any added value to using bi-specific nanobodies. Instead of testing the nano-Fcs at a concentration where all of them yield close to 100% inhibition, it may be useful to test the nano-Fcs at a range of concentrations to allow determination of an IC50. This may allow comparison of the potency of different nano-Fcs including the chimeric nanobodies and determine if the bi-specific nanobodies are more potent that mono-specific nanobodies.
