# Supplementary material for: Pfs48/45 nanobodies block Plasmodium falciparum transmission
Source: PLoS Pathog. 2026 Jan 27;22(1):e1013884. doi: 10.1371/journal.ppat.1013884 (PMC12858062; doi:10.1371/journal.ppat.1013884)
Supplement: Attachment — Submitted filename: Lyons_PLOSPathogens_Rebuttal.pdf [file ppat.1013884.s008.pdf]

WEHI
brighter together

The Walter and Eliza Hall Institute of Medical Research
ABN 12 004 251 423
1G Royal Parade Parkville Victoria 3052 Australia
T +61 3 9345 2555 F +61 3 9347 0852
www.wehi.edu.au

9th of September 2025

Dear Editor,

We are pleased about the possibility of submitting a revised manuscript. As you mention in the review decision letter, it will be important to address the dose-dependent study for the bispecific and we thank you for acknowledging how challenging such studies are (which they are!).

However, in response to the reviewers below, we provide reasons why we think that the dose-dependent studies are ***not*** required for the conclusions made in our paper. Our paper shows that you can use nanobodies and bispecific nanobody formats against Pfs48/45 to reduce transmission, which is the main purpose of the paper and these conclusions remain valid at the single concentration tested. As such, we strongly believe that doing a dose-dependent study for transmission blocking experiments with mosquitoes does not change this main conclusion.

More work in the future will be required to translate these nanobody modalities to be clinic ready. As such, it would be outside the scope of this manuscript to perform dose titration of the nanobody entities and would require too many resources for our current study.

Kind regards,

Wai-Hong Tham

Professor Wai-Hong Tham
The Walter + Eliza Hall Institute
m: + 61 430044842
w: https://www.wehi.edu.au/researcher/wai-hong-tham/

Reviewer #1
Part I - Summary

Reviewer #1: This is a well-conducted study in which the authors develop a panel of nanobodies targeting the transmission-blocking malaria vaccine candidate Pfs48/45. They find that some of these nanobodies can block the transmission of parasites to mosquitos. They determine the crystal structure of the best of these nanobodies bound to the D3 domain of Pfs48/45 and show that overlaps with the epitope of some, but not all transmission-blocking antibodies against domain D3. This study is of interest and, as explained in the discussion, the nanobodies might have some applications in addition to those found for the antibodies. It is therefore a worth-while study.

Response: We thank the reviewer for their kind comments.

The second part of the study combines the best nanobody from this study with a nanobody targeting a second transmission blocking vaccine immunogen Pfs230 as a bispecific. This is tested at single concentration and is as effective at blocking transmission as the two parental nanobodies. I found this part of the study quite hard to interpret as the concentration dependence of the bispecific was not compared with that of the monospecifics with TRAs just

done at one concentration at which there was very high TRA. It is therefore hard to conclude much from this part of the study. Line 288 stated that "bispecific nanobodies do not appear to have significantly higher transmission-blocking activity that B2-Fc" and I did not agree that the authors have the evidence for this statement either way. It is also the case that these were not compared with a monovalent nanobody and so it also can't be concluded that the two arms were both functional. More data is required here to draw clear conclusions from this finding.

Response: We agree with the reviewer that Line 288 requires some restating. We have modified this sentence on line 288 to be "…bispecific nanobodies have similar transmission-blocking activity as B2-Fc alone.", which most closely reflects the results.

We respectfully disagree with the reviewer that more data is required as our results in Figure 4B clearly shows that the bispecific binds to both recombinant Pfs230 and Pfs48/45 and blocks transmission in Figure 4C.

In general therefore, the first part of Pfs48/45 nAbs is clear and well done, but the section on the bispecific is hard to interpret and is less well controlled. The paper might be better without the bispecific section, allowing the authors to conduct a fully controlled study on these bispecifics.

Response: We do not expect that the bispecific would be more potent than mono-specific format. The bispecific format has additional advantages such as delivery of only one antibody to target two antigens. Our main purpose was to deliver a bispecific that clearly recognizes two different antigens while retaining comparable transmission blocking activity. Our results in Figure 4B and 4C have clearly shown that the bispecific format can block transmission and warrants inclusion into the manuscript.

We also respectfully disagree with the reviewer that the experiments are not well controlled. In Figure 4B, we determine the affinities of the monospecifics and bispecifics relative to the two antigens Pfs230 and Pfs48/45 and clearly show that the bispecifics can recognize both antigens in contrast to the monospecifics. For the transmission blocking experiments shown in Figure 4C, we control with a SARS-CoV-2 Nb-Fc which as expected does not block transmission and we have also included the transmission blocking monoclonal antibody TB31F as a control. We believe that all relevant controls have been added, and our results are valid.

Part II – Major Issues: Key Experiments Required for Acceptance
Reviewer #1: No experiments for the Pfs48/45 nanobody part.
For the bispecific, if this remains in the paper, it would be best to conduct the TRAs at different dilutions to know how it performs relative to monospecific and Fabs.

Response: We do not expect that the bispecific would be more potent than mono-specific format as it cannot bind the two antigens at the same time. Nevertheless, the bispecific format has additional advantages such as delivery of only one antibody to target two antigens. Our main purpose was to deliver a bispecific that clearly recognizes two different antigens while retaining comparable transmission blocking activity. Our results in Figure 4 have clearly shown that the bispecific format can bind to two different antigens and can block transmission. As such our conclusions are valid without the need to conduct the TRAs at different dilutions.

Part III – Minor Issues: Editorial and Data Presentation Modifications
Reviewer #1: (No Response)

Reviewer #2

In this manuscript the authors have explored the feasibility of developing nanobodies against the sexual stage antigens, Pfs48/45 and Pfs230, to inhibit transmission of P. falciparum. Following immunization of alpacas with deglycosylated Pfs48/45 D3 a nanobody phage library was generated and screened for binding to Pfs48/45 by ELISA. Anti-Pfs48/45 nanobodies were produced as recombinant proteins fused to Fc and tested for recognition of Pfs48/45 by ELISA and immunofluorescence assay (IFA). The interaction of three nano-Fcs, A4-Fc, B2-Fc and C3-Fc, that bind Pfs48/45 D3 domain was analyzed. The three nanobodies react to overlapping epitopes on Pfs48/45 D3 but don't inhibit binding of a previously identified mAb referred to as TB31F. The three nanobodies, A4-Fc, B2-Fc and C3-Fc, like TB31F, block parasite transmission with high efficiency. The binding site of one of the nanobodies, B2-Fc, was determined by solving the structure of the nanobody in a complex with Pfs48/45 D3. By linking previously identified nanobodies, F5 and F10, against Pfs230, with B2 the authors produced chimeric bispecific nanobodies, that react with both Pfs48/45 and Pfs230. Importantly, the chimeras retain the ability to target the mosquito stages and block transmission efficiently. The authors have shown elegantly the feasibility of using nanobodies to inhibit P. falciparum transmission. They should address the following main comment:

The characterization of the nanobodies in terms of binding to the antigens and mapping of the binding epitopes is clearly described. However, the function studies testing the ability of the nanobodies to block parasite transmission need additional work. The nanobodies were tested for inhibition of transmission at a single dilution at which close to 100% inhibition is achieved. It would have been informative to test the nano-Fcs at different concentrations to identify the most potent nano-bodies for transmission blocking activity.

Response: Reviewer 2 says " It would have been informative to test the nano-Fcs at different concentrations to identify the most potent nano-bodies for transmission blocking activity". However, in Figure 2C, at a single concentration of 100 ug/ml, we already observed that B2-Fc is more potent (99, 99% TRA) compared to A4-Fc (86, 89% TRA) and C3-Fc (79, 95% TRA). We also state on Line 204, "B2-Fc was the most potent nanobody, with a transmission-reducing activity of 99%...". As such, TRA at different concentration will not change our conclusions and our conclusion with a single concentration remains valid.

The experiment with bispecific nano-Fcs was also performed at a single concentration. At the concentration used even mono-specific nanobodies yield close to 100% inhibition. It is therefore difficult to determine if there is any added value to using bi-specific nanobodies.

Response: As mentioned above, we do not expect that the bispecific would be more potent than mono-specific format. The bispecific format has additional advantages such as delivery of only one antibody to target two antigens. Our results in Figure 4 have clearly shown that the bispecific format can bind to two different antigens and can block transmission. As such our conclusions are valid without the need to conduct the TRAs at different dilutions.

Instead of testing the nano-Fcs at a concentration where all of them yield close to 100% inhibition, it may be useful to test the nano-Fcs at a range of concentrations to allow

determination of an IC50. This may allow comparison of the potency of different nano-Fcs including the chimeric nanobodies and determine if the bi-specific nanobodies are more potent that mono-specific nanobodies.

Response:
The main purpose of our paper is to show that you can use nanobodies and bispecific nanobody formats against Pfs48/45 to reduce transmission and these conclusions remain valid at the single concentration tested. As such, we strongly believe that doing a dose-dependent study for transmission blocking experiments with mosquitoes does not change this main conclusion.

In addition, there are many published papers that only use one concentration of antibody in transmission-blocking experiments. From these papers, we also note that our use of 100 ug/ml as a concentration is well within range of standard experiments and is not too high a concentration.
I list the papers below:
- Canepa, G.E., Molina-Cruz, A., Yenkoidiok-Douti, L. et al. Antibody targeting of a specific region of Pfs47 blocks Plasmodium falciparum malaria transmission. npj Vaccines 3, 26 (2018). https://doi.org/10.1038/s41541-018-0065-5(Figure 3a, b, c, mAbs tested at 200 ug/ml)
- Singh, K., Burkhardt, M., Nakuchima, S. et al. Structure and function of a malaria transmission blocking vaccine targeting Pfs230 and Pfs230-Pfs48/45 proteins. Commun Biol 3, 395 (2020). https://doi.org/10.1038/s42003-020-01123-9 (Table 1, mAbs tested at 1 mg/ml)
- Tang, W.K., Coelho, C.H., Miura, K. et al. A human antibody epitope map of Pfs230D1 derived from analysis of individuals vaccinated with a malaria transmission-blocking vaccine. Immunity 56, 2 (2023) https://doi.org/10.1016/j.immuni.2023.01.012 (Figure 1, mAbs tested at 100 ug/ml)
- Miura, K., Jongert, E., Deng, B. et al. Effect of ingested human antibodies induced by RTS, S/AS01 malaria vaccination in children on Plasmodium falciparum oocyst formation and sporogony in mosquitoes. Malar J 13, 263 (2014). https://doi: 10.1186/1475-2875-13-263.(Tables 4, 5 and 6, mAbs tested at 231 ug/mL)
