# Supplementary material for: Pfs48/45 nanobodies block Plasmodium falciparum transmission
Source: PLoS Pathog. 2026 Jan 27;22(1):e1013884. doi: 10.1371/journal.ppat.1013884 (PMC12858062; doi:10.1371/journal.ppat.1013884)
Supplement: Attachment — Submitted filename: Lyons_021125_PPRebuttal_Final.pdf [file ppat.1013884.s009.pdf]

WEHI
brighter together

The Walter and Eliza Hall Institute of Medical Research
ABN 12 004 251 423
1G Royal Parade Parkville Victoria 3052 Australia
T +61 3 9345 2555 F +61 3 9347 0852
www.wehi.edu.au

5th of November 2025

Dear Editors,

We are pleased about the possibility of submitting a revised manuscript and the recognition by all reviewers that nanobodies and bispecifics against Pfs48/45 are novel for the field of transmission blocking interventions. We have addressed all reviewers' comments below.

In the initial review decision letter, while the reviewers have asked for the dose-dependent study for the bispecific, we thank you for acknowledging how challenging such studies are (which they are!).

In response to the reviewers below, we provide reasons why we think that the dose-dependent studies are ***not*** required for the conclusions made in our paper. Our paper shows that you can use nanobodies and bispecific nanobody formats against Pfs48/45 to reduce transmission, which is the main purpose of the paper and these conclusions remain valid at the single concentration tested. As such, we strongly believe that doing a dose-dependent study for transmission blocking experiments with mosquitoes does not change this main conclusion.

More work will be required in the future to translate these nanobody modalities to be clinic ready. As such, it would be outside the scope of this manuscript to perform dose titration of the nanobody entities and would require too many resources for our current study.

Kind regards,

Wai-Hong Tham

Professor Wai-Hong Tham
The Walter + Eliza Hall Institute
m: + 61 430044842
w: https://www.wehi.edu.au/researcher/wai-hong-tham/

Reviewers' Comments:
Part I - Summary
Please use this section to discuss strengths/weaknesses of study, novelty/significance, general execution and scholarship.

Reviewer #1: This is a well-conducted study in which the authors develop a panel of nanobodies targeting the transmission-blocking malaria vaccine candidate Pfs48/45. They find that some of these nanobodies can block the transmission of parasites to mosquitos. They determine the crystal structure of the best of these nanobodies bound to the D3 domain of Pfs48/45 and show that overlaps with the epitope of some, but not all transmission-blocking antibodies against domain D3. This study is of interest and, as explained in the discussion, the nanobodies might have some applications in addition to those found for the antibodies. It is therefore a worth-while study.
Response: We thank the reviewer for their kind comments.

The second part of the study combines the best nanobody from this study with a nanobody targeting a second transmission blocking vaccine immunogen Pfs230 as a bispecific. This is tested at single concentration and is as effective at blocking transmission as the two parental nanobodies. I found this part of the study quite hard to interpret as the concentration dependence of the bispecific was not compared with that of the monospecifics with TRAs just done at one concentration at which there was very high TRA. It is therefore hard to conclude much from this part of the study. Line 288 stated that "bispecific nanobodies do not appear to have significantly higher transmission-blocking activity that B2-Fc" and I did not agree that the authors have the evidence for this statement either way. It is also the case that these were not compared with a monovalent nanobody and so it also can't be concluded that the two arms were both functional. More data is required here to draw clear conclusions from this finding.
Response: We agree with the reviewer that Line 293 requires restating. We have modified this sentence on line 292 to be "…bispecific nanobodies have similar transmission-blocking activity as B2-Fc alone.", which most closely reflects the results.

We respectfully disagree with the reviewer that more data is required as our results in Figure 4B clearly shows that the bispecific binds to both recombinant Pfs230 and Pfs48/45 and blocks transmission in Figure 4C.

In general therefore, the first part of Pfs48/45 nAbs is clear and well done, but the section on the bispecific is hard to interpret and is less well controlled. The paper might be better without the bispecific section, allowing the authors to conduct a fully controlled study on these bispecifics.
Response: We do not expect that the bispecific would be more potent than mono-specific format. The bispecific format has additional advantages such as delivery of only one antibody to target two antigens. Our main purpose was to deliver a bispecific that clearly recognizes two different antigens while retaining comparable transmission blocking activity. Our results in Figure 4B and 4C have clearly shown that the bispecific format can block transmission and warrants inclusion into the manuscript.

We also respectfully disagree with the reviewer that the experiments are not well controlled. In Figure 4B, we determine the affinities of the monospecifics and bispecifics relative to the two antigens Pfs230 and Pfs48/45 and clearly show that the bispecifics can recognize both antigens in contrast to the monospecifics. For the transmission blocking experiments shown in Figure 4C, we control with a SARS-CoV-2 Nb-Fc which as expected does not block transmission and we have also included the transmission blocking monoclonal antibody TB31F as a control. We believe that all relevant controls have been added, and our results are valid.

Reviewer #2:
Lyons et al. develop the first set of nanobodies against the Plasmodium falciparum surface protein Pfs48/45, one of the leading targets for transmission-blocking vaccine development. They identify a set of three nanobodies that bind to Pfs48/45 domain 3 with nanomolar affinities and all bind the same epitope. They assess their transmission-blocking potential in standard membrane feedings assays (SMFAs), showing that these nanobodies exhibit high TRA at 100ug/mL. However, since the nanobodies were only tested at a single high dose, it remains unclear how potent these antibodies really are and thus compare to previously reported monoclonal antibodies. Furthermore, they solved the structure of the most potent nanobody B2 in complex with Pfs48/45 domain 3 allowing them to identify the B2 epitope. This B2 epitope

largely overlaps with the previously reported epitope of potent human antibodies RUPA-44 and RUPA-117. The authors continue with the most promising nanobody to develop two bispecific nanobodies, that combine nanobody B2 with variable domains of nanobodies targeting TBV candidate Pfs230D1. They show that these bispecific nanobodies can still bind both targets and block transmission at 100 ug/mL. However, the design of their SMFA experiment could not show how the potency of the bi-specific nanobodies compare to the parental nanobodies and thus whether there is synergistic/additive/no/negative effect when combining two specificities in one molecule.

The novelty of this study is that it describes the first nanobodies against Pfs48/45. These may indeed be useful tools for studying biology and generating parasite-resistant mosquitoes, though it is more difficult to see how these (non-human) nanobodies can become attractive for prophylactic use in humans in the near future. Another novelty described in this study is the generation and testing of bi-specific antibodies against TBV candidates, but more functional assays are required to assess their value.

The isolation, binding assessment and structural analyses of the nanobodies are well executed and described. The major weakness of this study is that functional characterisation is very limited and provides little insight in the actual potency of (bi-specific) nanobodies. More in-depth functional analysis is required to understand the potential value of these nanobodies, see below.

Response: We thank the reviewer for acknowledging that "The novelty of this study is that it describes the first nanobodies against Pfs48/45.". Indeed, the main purpose of our paper is to show that you can use nanobodies and bispecific nanobody formats against Pfs48/45 to reduce transmission and these conclusions remain valid at the single concentration tested. As such, we strongly believe that doing a dose-dependent study for transmission blocking experiments with mosquitoes does not change this main conclusion.

Reviewer #3: Malaria transmission-blocking vaccines (TBVs) are crucial strategies for eliminating malaria, with Pfs48/45 and Pfs230 being the primary vaccine targets. The authors developed nanobodies against Pfs48/45 that recognize gametocytes and exhibit strong transmission-reducing activity. The crystal structure of the most potent nanobody bound to the recombinant Pfs48/45 D3 revealed that it attaches to a different epitope than the known human mAb, TB31F. Importantly, they also created bispecific nanobodies capable of targeting both Pfs48/45 and Pfs230, fused to a human Fc domain. These bispecific nanobodies recognize both Pfs48/45 and Pfs230, thereby reducing malaria transmission.

Very interesting and important work. Establishing bispecific nanobodies targeting both Pfs48/45 and Pfs230 could serve as an alternative approach to current TBV development efforts, supporting malaria elimination. All experiments are carefully designed and carried out to a high standard by leading experts in this field of research. I have some comments to further improve this manuscript.
Response: We thank the reviewer for their kind comments.

Part II – Major Issues: Key Experiments Required for Acceptance

Reviewer #1: No experiments for the Pfs48/45 nanobody part.

For the bispecific, if this remains in the paper, it would be best to conduct the TRAs at different dilutions to know how it performs relative to monospecific and Fabs.
Response: We do not expect that the bispecific would be more potent than mono-specific format as it cannot bind the two antigens at the same time. Nevertheless, the bispecific format has additional advantages such as delivery of only one antibody to target two antigens. Our main purpose was to deliver a bispecific that clearly recognizes two different antigens while retaining comparable transmission blocking activity. Our results in Figure 4 have clearly shown that the bispecific format can bind to two different antigens and can block transmission. As such our conclusions are valid without the need to conduct the TRAs at different dilutions.

Reviewer #2: • Figure 2: The functional assessment of the nanobodies should be expanded with titrations of the nanobodies. The nanobodies should be diluted till TRA is lost or drops below 50-80% to allow estimation of their IC80 value and comparison with other antibodies described in literature.
Response: The main purpose of our paper is to show that you can use nanobodies and bispecific nanobody formats against Pfs48/45 to reduce transmission and these conclusions remain valid at the single concentration tested. As such, we strongly believe that doing a dose-dependent study for transmission blocking experiments with mosquitoes does not change this main conclusion.

In addition, there are many published papers that only use one concentration of antibody in transmission-blocking experiments. From these papers, we also note that our use of 100 ug/ml as a concentration is well within range of standard experiments and is not too high a concentration.
I list the papers below:

- Canepa, G.E., Molina-Cruz, A., Yenkoidiok-Douti, L. et al. Antibody targeting of a specific region of Pfs47 blocks Plasmodium falciparum malaria transmission. npj Vaccines 3, 26 (2018). https://doi.org/10.1038/s41541-018-0065-5(Figure 3a, b, c, mAbs tested at 200 ug/ml)
- Singh, K., Burkhardt, M., Nakuchima, S. et al. Structure and function of a malaria transmission blocking vaccine targeting Pfs230 and Pfs230-Pfs48/45 proteins. Commun Biol 3, 395 (2020). https://doi.org/10.1038/s42003-020-01123-9 (Table 1, mAbs tested at 1 mg/ml)
- Tang, W.K., Coelho, C.H., Miura, K. et al. A human antibody epitope map of Pfs230D1 derived from analysis of individuals vaccinated with a malaria transmission-blocking vaccine. Immunity 56, 2 (2023) https://doi.org/10.1016/j.immuni.2023.01.012 (Figure 1, mAbs tested at 100 ug/ml)
- Miura, K., Jongert, E., Deng, B. et al. Effect of ingested human antibodies induced by RTS, S/AS01 malaria vaccination in children on Plasmodium falciparum oocyst formation and sporogony in mosquitoes. Malar J 13, 263 (2014). https://doi: 10.1186/1475-2875-13-263.(Tables 4, 5 and 6, mAbs tested at 231 ug/mL)

• Figure 4: The bispecific nanobodies show very high TRA, but so does the parental nanobody B2-Fc. The TRA is almost saturated making it impossible to compare the potency of the (bi-specific) nanobodies. Side-by-side titrations of the (parental) nanobodies and bispecific nanobodies should be conducted to be able to assess whether the bispecific nanobodies are more potent, similarly potent, or less potent than their parental counterparts. Note that the parental nanobodies F5 and F10 should also be included in these experiments. If bispecific

nanobodies turn out to be more potent than their parental counterparts, the authors could test whether this can be explained by assessing whether bispecifics have a higher apparent affinity to gametes.
Response: Please refer to the response above on the main purpose of the paper and we feel that the titrations are outside the scope of this manuscript.

Reviewer #3: 1) Line 189, Figure 2B
The co-localization of TB31F and three nanobodies' staining is clear. However, the lattice-like staining pattern was unusual because Pfs48/45 is located on the surface of gametocytes or gametes. I believe that the membrane structure of the gametocyte antigen was damaged during sample preparation, including fixation. Therefore, I strongly recommend the authors repeat the IFA experiment using another fixation method, such as PFA fixation, which is commonly used in other studies.
Response: As recommended by the reviewer, we have repeated the immunofluorescence assay of stage V gametocytes using PFA fixation. Using PFA, the lattice-like pattern was not observed while nanobody-Fc staining and co-localization with anti-Pfs48/45 mAb TB31F was still apparent. Thank you for the suggestion!

We have modified the following sections:
Figure 2B: The panel of images have been replaced with PFA-fixed samples.

Results, line 190**:** We have removed the following statement from the previous version "and produces the same lattice-like pattern of staining on the gametocytes" and the sentence now reads: "The staining of all three anti-Pfs48/45 nanobody-Fcs colocalises with that of TB31F (Fig 2B)."

Methods, Immunofluorescence assay, line 572: We have included text that describes the PFA fixation method we used.

2) Lines 255-264, SMFA of bispecific nanobody-Fcs, Figure 4C
It is crucial to compare the potency of mono- and bispecific nanobodies. Therefore, I strongly recommend that the authors add dose-response SMFA to calculate the IC50. Then they can compare the potencies.
Response: The main purpose of our paper is to show that you can use nanobodies and bispecific nanobody formats against Pfs48/45 to reduce transmission and these conclusions remain valid at the single concentration tested. As such, we strongly believe that doing a dose-dependent study for transmission blocking experiments with mosquitoes does not change this main conclusion.

We do not expect that the bispecific would be more potent than mono-specific format as it cannot bind the two antigens at the same time. Nevertheless, the bispecific format has additional advantages such as delivery of only one antibody to target two antigens. Our main purpose was to deliver a bispecific that clearly recognizes two different antigens while retaining comparable transmission blocking activity. Our results in Figure 4 have clearly shown that the bispecific format can bind to two different antigens and can block transmission. As such our conclusions are valid without the need to conduct the TRAs at different dilutions.

3) Lines 1000-1001, S1 Fig A

Some additional bands are still visible in the post-treatment lanes. Please estimate the purity by densitometry.

Response: As requested by the reviewer, we have used densitometry to estimate the purity. Our densitometry approach is shown in Rebuttal Figure 1. In summary, the peak for glycosylated bands in reducing and non-reducing conditions is not present after PNGaseF treatment (Peaks a and d are missing from Lanes 3 and 4). In addition, our deglycoslyated Pfs48/45 D3 was used for crystallization purposes and therefore we maintain that the purification was pure.

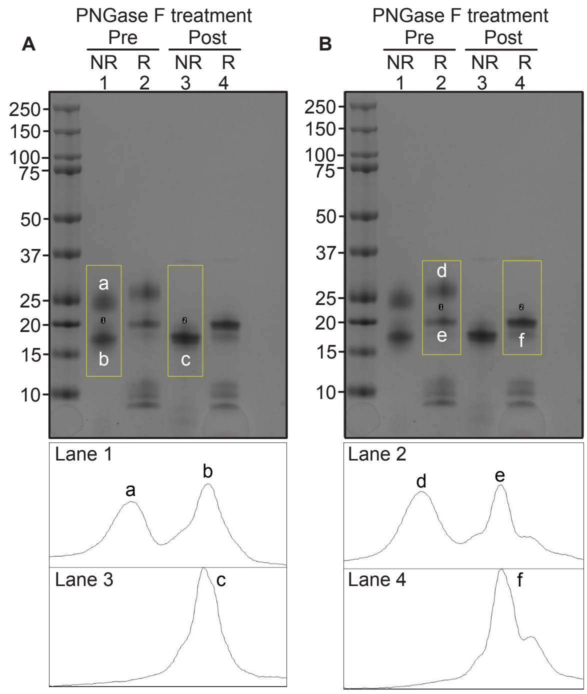

Rebuttal Figure 1. Densitometry of purified Pfs48/45 D3 pre- and post- PNGase F treatment from Figure S1.

(A) Densitometry analysis of non-reducing (NR) samples of purified Pfs48/45 D3. Analysis was done on the regions highlighted by yellow boxes using FIJI (Image J2, Version: 2.14.0/1.5h) and the inbuilt gel analysis tool. Data for each lane was plotted, showing the presence of two peaks in the pre-PNGase treated sample (lane 1), and a single peak post treatment (lane 3), indicating no glycosylated forms are present post treatment. (B) Densitometry analysis of reducing (R) c samples of purified Pfs48/45 D3. Analysis was done as described above. Data for each lane was plotted, showing the presence of two peaks in the pre-PNGase treated sample (lane 2), and a single peak post treatment (lane 4), indicating no glycosylated forms are present post treatment. Bands in the gel have been labelled above or below the band, and labels correspond to the peaks seen in the plots.

Part III – Minor Issues: Editorial and Data Presentation Modifications

Please use this section for editorial suggestions as well as relatively minor modifications of existing data that would enhance clarity.

Reviewer #1: (No Response)

Reviewer #2: • Line 27: "…reveals it binds a distinct epitope to TB31F, a leading transmission-blocking monoclonal antibody." This phrasing suggests that the nanobody B2 binds a novel epitope on the Pfs48/45 surface, while it largely overlaps with the previously characterized RUPA-44 and RUPA-117 epitope. Please refer to the RUPA-44/RUPA-117 epitope in the abstract.

Response: We have added the following text in line 27 in the abstract to refer to the RUPA-44 and RUPA-117 epitopes: "The crystal structure of our most potent nanobody in complex with Pfs48/45 reveals it binds a distinct epitope to TB31F, a leading transmission-blocking monoclonal antibody but *to similar epitopes as RUPA-44 and RUPA-117*."

• Line 73: Please mention the extended conformation observed by cryo-EM as observed by Kucharska et al. (2025).

Response: We have modified the sentence to read, line 72: "Crystal structures and cryo-EM structures of Pfs48/45 indicate that the three 6-cysteine domains can adopt a planar triangular arrangement on the parasite surface as well as an extended conformation [7,8].

• Line 86: In the same paper, by Kucharska et al., the authors have characterized the epitopes of mAbs RUPA-58 and RUPA-154, which bind to D1 and D2, respectively. Include these in the description here.

Response: We have modified the sentence in line 85 to include the recently characterized epitopes of mAbs RUPA-58 (D1-specific), RUPA-154 (D2-specific) by Kucharska et al, and RUPA-71 (D1-specific) by Bekkering, Yoo, Hailemariam et al. This sentence now reads, line 86:

"Crystal structures and cryo-electron microscopy (cryo-EM) structures for eleven transmission blocking mAbs in complex with Pfs48/45 are available, of which seven bind to D3, two to D2 and two to D1 [7,8,30–32,34,35]."

• Lines 156-160: Can the authors speculate why some nanobodies show binding by Western Blot and ELISA, but not by BLI?

Response: Both Western blotting and ELISA can denature the antigen due to the SDS and hydrophobic surface coating respectively, and present linear epitopes that are not present when the protein is in solution in BLI assays.

• Line 160: Figure S3 mentions Kd = 3.3 nM, not 3.8 nM.

Response: The value of 3.8 nM is stated in Figure 1F and line 162 is correct. It represents the K_D value of two BLI replicates. In Figure S3, the value of K_D 3.3 nM is referring to the single representative BLI curve that we have shown in Figure S3.

• Line 212-215: Please include a supplementary figure showing the hydrogen/ionic interactions between Pfs48/45 D3 and B2

Response: As requested, we have added a panel B to supplementary figure S3 to show the hydrogen bonds and ionic interactions between Pfs48/45 and nanobody B2. The figure legend to Figure S3 has also been modified accordingly.

• Figure 3C: it would be more meaningful to replace the figure with the non-overlapping RUPA-29 epitope with a figure showing the RUPA-117 epitope, which largely overlaps with B2 and RUPA-44.

Response: We agree with the reviewer and as requested, in Figure 3C, we have replaced the figure with the non-overlapping RUPA-29 epitope with a figure showing the RUPA-117 epitope, which largely overlaps with nanobody B2. We have modified the figure legend and results as listed below.

Line 222: In addition, we compared the binding epitope of B2 with those of previously published anti-Pfs48/45 D3 antibodies TB31F (PDB ID 6E63), 32F3 (PDB ID 7ZWI), RUPA-117 (PDB ID 7UNB) and RUPA-44 (PDB ID 7UXL) (Fig 3C).

Line 227: The B2 epitope overlaps with that of 32F3 by two residues and with those of RUPA-117 and RUPA-44 by 13 residues (Fig 3C, bottom row).

Figure legend 3C, line 953:
(C) Pfs48/45 with binding epitopes of nanobody B2 and transmission-blocking antibodies TB31F (PDB ID 6E63), 32F3 (PDB ID 7ZWI), RUPA-117 (PDB ID 7UNB) and RUPA-44 (PDB ID 7UXL), which have previously been crystallised in complex with Pfs48/45.

• Figure 4B: the mentioned affinities do not correspond to the ones found in the text or Figure S4C.
Response: The affinities in the text correspond to the values quoted in Figure S4C, which are the average affinities from two replicates. These values are accurate. Figure 4B shows a representative experiment and the value quoted is for that specific replicate.

• Line 253-254: Please change Pfs230 to Pfs230 D1 and Pfs48/45 to Pfs48/45 D3.
Response: We have modified two sentences within this paragraph to refer to Pfs230 D1 and Pfs48/45 D3.

Line 251: "As expected, we observed that B2-Fc bound to Pfs48/45 D3 but not Pfs230 D1, while F5-Fc and F10-Fc bound to Pfs230 D1 but not Pfs48/45 D3 (Fig 4B and S4C)."

Line 257: "These results show that the bispecific nanobodies bind both Pfs230 D1 and Pfs48/45 D3 with similar affinities to the individual nanobody-Fcs."

• Line 286: We agree that based on the reported structures, co-binding on the same complex is unlikely. However, since Pfs48/45 and Pfs230 are highly expressed, it does not seem impossible for the same nanobody to bind two different complexes at the same time.
Response: We do not disagree with the reviewer on the statement on co-binding of the same complex so we have left the sentence as is.

• Line 288: "… which may explain why the bispecific nanobodies do not appear to have a signficantly higher transmission-blocking activity." Please update this statement following the outcome of the requested SMFA titration experiments.
Response: We agree with the reviewer that Line 292 requires some restating. We have modified this sentence on line 292 to be "…bispecific nanobodies have similar transmission-blocking activity as B2-Fc alone.", which most closely reflects the results.

We respectfully disagree with the reviewer that more data is required as our results in Figure 4B clearly shows that the bispecific binds to both recombinant Pfs230 and Pfs48/45 and blocks transmission in Figure 4C.

• Line 305 – 318: the supposed advantages of using a nanobody compared to the already humanized TB31F or one of the previously characterized human-derived monoclonal antibodies, which would not suffer from the mentioned setbacks, is not made clear in the context of clinical use.
Response: We agree that humanized TB31F is suitable for clinical use and have stated so. The main purpose of the paragraph was to describe what else needed to occur for the translational of nanobody therapeutics and as such we maintain the paragraph as is.

• Line 328: There are more examples of expressing single chain monoclonal antibodies against mosquito-stages, including Green et al. (2023) which use anti-CSP antibody sc2A10 to block transmission by targeting hemolymph sporozoites.

Response: Thank you for highlighting a missing reference. We have added the following text for this study, line 336: "Transgenic mosquitoes expressing single-chain version of antibody 2A10, which binds sporozoites of the malaria parasite *Plasmodium falciparum,* showed a reduction in transmission of *Plasmodium berghei* expressing the *P. falciparum* circumsporozoite protein to mice [61]."

• Line 583: it would be helpful to explicitly mention that human serum contained active human complement (assuming it did).

Response: We use heat inactivated human serum and have now added in the follow text, line 594: "Blood meals were comprised of 300 µL heat inactivated O+ human serum and 200 µL O+ erythrocytes with a stage V gametocytaemia of ~0.5%."

• Figure legends 2d and 4c: please include antibody/nanobody concentrations in the figure legends and specify whether complement was added.

Response: As requested, we have included the following sentences into the respective legends. Figure legend 2c: Blood meals included heat-inactivated human serum, 50 µg of nanobody-Fcs or 100 µg TB31F in PBS or 10 µL PBS.

Figure 4c: Blood meals included heat inactivated human serum, 50 µg of bispecifics or 100 µg TB31F in PBS or 10 µL PBS.

Reviewer #3: 1) Line 318 "humanisation of the nanobody backbone [55] would need to be considered."

I agree with this statement because this step is crucial for developing this bispecific new intervention to move toward clinical development. When I read reference #55, humanization of camelid Nanobodies is possible but not straightforward. Please cite a reference that details the clinical development of nanobody-based human antibodies (as an example) if available.

Response: Unfortunately, while there are some humanized nanobodies in clinical development, there are no publications that details their clinical development or "humanization". As such we propose that our current reference Vincke et al is the most appropriate.

2) Recombinant Pfs48/45 D3 expression

Why did the Authors use two different methodologies to express "Pfs48/45 D3 (Sf21)" and "stabilized Pfs48/45 D3 (Expi293)"? Please explain.

Response: For Pfs48/45 D3, we used insect cell expression (Sf21) as it is our laboratory's standard expression system for 6-cysteine proteins. For stabilized Pfs48/45 D3, we used the construct described in McLeod et al, Immunity 2022 and as such we followed the protocol in Expi293 which is described in their paper.

3) Others:

Line 283: "in two preprints" -> The Author's group paper has been published in Science recently.

Response: We have now added the correct references, line 287: "Recently, the structure of the endogenous Pfs48/45-Pfs230 complex has been described [35,52]."

Line 393: "GERBU FAMA" -> Please add company information.
Response: We have added the company information, line 399: "One alpaca was immunised six times with ~150 µg of recombinant Pfs48/45 D3 using GERBU FAMA (GERBU Biotechnik) as an adjuvant."

Line 537: "molar ratio of 1:1.5" -> Why not 1:1? Please explain.
Response: This is the standard protocol in our lab to ensure that we have full binding of Pfs48/45 D3 by using an excess amount of nanobody. Unbound nanobody is then separated out using size exclusion chromatography.

Line 586: "Blood-feeding….for 1-2 h" -> Why so long? Usually, the mosquitoes become full-stomach in 30 minutes.
Response: This is our standard protocol in our insectary with most feeding times closer to an hour. More importantly, all mosquitoes were treated the same for the feeding duration for their respective SMFA.

Line 948: Figure 1G Why are non-competitive wells higher than 100%? Please explain.
Response: In our BLI competition setup, one nanobody is immobilized and then dipped into wells containing either Pfs48/45 D3 alone or Pfs48/45 D3+nanobody/Fab. The Pfs48/45 D3 alone is our baseline no competition (100%) and the Pfs48/45 D3+nanobody/Fab percent competition is calculated relative to this. The BLI signal is affected by the size of the protein binding. If a nanobody or Fab is not competing, the BLI signal will correspond to Pfs48/45 D3+nanobody/Fab. Since Pfs48/45 D3 is only 16 kDa, the addition of the nanobody/Fab results in a larger BLI signal compared to Pfs48/45 D3 alone, which explains why the non-competitive wells are higher than 100%.

PDF attachment by Reviewer #2
In this manuscript the authors have explored the feasibility of developing nanobodies against the sexual stage antigens, Pfs48/45 and Pfs230, to inhibit transmission of P. falciparum. Following immunization of alpacas with deglycosylated Pfs48/45 D3 a nanobody phage library was generated and screened for binding to Pfs48/45 by ELISA. Anti- Pfs48/45 nanobodies were produced as recombinant proteins fused to Fc and tested for recognition of Pfs48/45 by ELISA and immunofluorescence assay (IFA). The interaction of three nano-Fcs, A4-Fc, B2-Fc and C3-Fc, that bind Pfs48/45 D3 domain was analyzed. The three nanobodies react to overlapping epitopes on Pfs48/45 D3 but don't inhibit binding of a previously identified mAb referred to as TB31F. The three nanobodies, A4-Fc, B2-Fc and C3-Fc, like TB31F, block parasite transmission with high efficiency. The binding site of one of the nanobodies, B2-Fc, was determined by solving the structure of the nanobody in a complex with Pfs48/45 D3. By linking previously identified nanobodies, F5 and F10, against Pfs230, with B2 the authors produced chimeric bispecific nanobodies, that react with both Pfs48/45 and Pfs230. Importantly, the chimeras retain the ability to target the mosquito stages and block transmission efficiently. The authors have shown elegantly the feasibility of using nanobodies to inhibit P. falciparum transmission.
Response: We thank the reviewer for a good summary of our manuscript.

They should address the following main comment:
The characterization of the nanobodies in terms of binding to the antigens and mapping of the binding epitopes is clearly described. However, the function studies testing the ability of the

nanobodies to block parasite transmission need additional work. The nanobodies were tested for inhibition of transmission at a single dilution at which close to 100% inhibition is achieved. It would have been informative to test the nano-Fcs at different concentrations to identify the most potent nano-bodies for transmission blocking activity. The experiment with bispecific nano-Fcs was also performed at a single concentration. At the concentration used even mono-specific nanobodies yield close to 100% inhibition. It is therefore difficult to determine if there is any added value to using bi-specific nanobodies. Instead of testing the nano-Fcs at a concentration where all of them yield close to 100% inhibition, it may be useful to test the nano-Fcs at a range of concentrations to allow determination of an IC50. This may allow comparison of the potency of different nano-Fcs including the chimeric nanobodies and determine if the bi-specific nanobodies are more potent that mono-specific nanobodies.

Response: The main purpose of our paper is to show that you can use nanobodies and bispecific nanobody formats against Pfs48/45 to reduce transmission and these conclusions remain valid at the single concentration tested. As such, we strongly believe that doing a dose-dependent study for transmission blocking experiments with mosquitoes does not change this main conclusion.

In addition, there are many published papers that only use one concentration of antibody in transmission-blocking experiments. From these papers, we also note that our use of 100 ug/ml as a concentration is well within range of standard experiments and is not too high a concentration.

I list the papers below:

- Canepa, G.E., Molina-Cruz, A., Yenkoidiok-Douti, L. et al. Antibody targeting of a specific region of Pfs47 blocks Plasmodium falciparum malaria transmission. npj Vaccines 3, 26 (2018). https://doi.org/10.1038/s41541-018-0065-5(Figure 3a, b, c, mAbs tested at 200 ug/ml)
- Singh, K., Burkhardt, M., Nakuchima, S. et al. Structure and function of a malaria transmission blocking vaccine targeting Pfs230 and Pfs230-Pfs48/45 proteins. Commun Biol 3, 395 (2020). https://doi.org/10.1038/s42003-020-01123-9 (Table 1, mAbs tested at 1 mg/ml)
- Tang, W.K., Coelho, C.H., Miura, K. et al. A human antibody epitope map of Pfs230D1 derived from analysis of individuals vaccinated with a malaria transmission-blocking vaccine. Immunity 56, 2 (2023) https://doi.org/10.1016/j.immuni.2023.01.012 (Figure 1, mAbs tested at 100 ug/ml)
- Miura, K., Jongert, E., Deng, B. et al. Effect of ingested human antibodies induced by RTS, S/AS01 malaria vaccination in children on Plasmodium falciparum oocyst formation and sporogony in mosquitoes. Malar J 13, 263 (2014). https://doi: 10.1186/1475-2875-13-263.(Tables 4, 5 and 6, mAbs tested at 231 ug/mL)
