# Supplementary material for: Pfs48/45 nanobodies block Plasmodium falciparum transmission
Source: PLoS Pathog. 2026 Jan 27;22(1):e1013884. doi: 10.1371/journal.ppat.1013884 (PMC12858062; doi:10.1371/journal.ppat.1013884)
Supplement: Attachment — Submitted filename: Lyons_Jan26_PPRebuttal.pdf [file ppat.1013884.s010.pdf]

WEHI
brighter together

The Walter and Eliza Hall Institute of Medical Research
ABN 12 004 251 423
1G Royal Parade Parkville Victoria 3052 Australia
T +61 3 9345 2555 F +61 3 9347 0852
www.wehi.edu.au

2nd January 2026

Dear Prof. Tracey Lamb,

We are pleased about the possibility of submitting a revised manuscript. We thank the reviewers for their thorough consideration of our previous revised manuscript and for providing the much-needed guidance for acceptance. We have addressed all reviewers' comments below.

Kind regards,

Wai-Hong Tham

Professor Wai-Hong Tham
The Walter + Eliza Hall Institute
m: + 61 430044842
w: https://www.wehi.edu.au/researcher/wai-hong-tham/

Additional Editor Comments:

Please address the comments of reviewer 1- summary section.
Response: As requested by the Editor, we have addressed the comments of Reviewer 1 in the Summary section and decided to remove the bispecific nanobody results which the reviewer has commented "would be recommending "accept" for that" for the rest of the manuscript as it stands.

Reviewers' Comments:

Reviewer's Responses to Questions
Part I - Summary
Please use this section to discuss strengths/weaknesses of study, novelty/significance, general execution and scholarship.

Reviewer #1: This is a tricky one. All three reviewers share the view that the authors should do a dose response titration for their bispecific antibody to show if they are or are not an improvement on the monospecific antibodies.

Reviewer 1: "it would be best to conduct the TRAs at different dilation"
Reviewer 2: "more in depth functional analysis is required to understand the potential value of these (bispecific) nanobodies"
Reviewer 3 " it is crucial to compare the potency....I recommend that the authors add dose-response SMFA data'

But the authors assert that this is outside the scope of the study as there was never an intention that the bispecific would be better.

Personally I don't understand the point of making a bispecific, which is going to bind to both antigens if the technical part is done correctly, without testing if it is better than monospecific. They have made the reagent, but not determined whether it is useful. I don't understand how they can assert that testing if it is better or not is outside the scope of the study. Surely this is the entire point of the study??

Personally I would support removing the bispecific and publishing the nanobody study, which is well done and suitable for publication. I would be recommending "accept" for that. They could then do the dose titration for the bispecific and publish a second paper on it.

I suppose, and I do generally support the right of authors to publish what they want if correctly done, that if they are determined, they could include the bispecific production. But then they should be very explicit in the paper that this has not been tested to the degree that they know if it is better than the monospecific and therefore can't make any comment on whether it is an improvement. Although from my perspective, this would seem unusual.
Response: We thank the reviewer for stating their considerations of the manuscript so clearly. Since they would be recommending "accept" for the manuscript if we remove the bispecific data, we have agreed to do so.

Below are our changes to remove the bispecific nanobody results:

a. Removal of Figure 4 and its associated legend.
b. Removal of Figure S4 and its associated legend.
c. Removal of the mention of bispecific nanobodies from the Abstract.
d. Removal of the mention of bispecific nanobodies from the Author Summary.
e. Removal of the bispecific nanobodies from the Results section
f. Removal of the methods associated with the generation of the bispecific nanobodies from the Material and Methods section.
g. Removal of the discussion of the bispecific nanobodies from the Discussion section.

Reviewer #2: I would like to thank the authors for addressing some of my comments, though the major ones still need to be addressed, see below.
Response: Thank you for your thorough consideration of our revision.

Reviewer #3: Although all the reviewers commented on the dose response SMFA, the Authors' rebuttal is reasonable.
I think the other comments are appropriately addressed.
Response: We thank the reviewer for acknowledging that the comments have been appropriately addressed.

Part II – Major Issues: Key Experiments Required for Acceptance
Please use this section to detail the key new experiments or modifications of existing experiments that should be absolutely required to validate study conclusions.

Generally, there should be no more than 3 such required experiments or major modifications for a "Major Revision" recommendation. If more than 3 experiments are necessary to validate the study conclusions, then you are encouraged to recommend "Reject".

Reviewer #1: (No Response)

Reviewer #2: I strongly disagree with the authors on their suggestion that titrations are not needed. They present their newly discovered and developed nanobodies as versatile antibodies that can reduce malaria transmission (also as concluding sentence of their abstract). It will therefore be important to titrate the nanobodies to understand (i) how potent these new nanobodies actually are and how that roughly compares to the many (human) monoclonal antibodies that have been described for Pfs48/45 so far and (ii) whether their bispecific nanobodies are less, equally or even more potent than the corresponding monospecific nanobodies.

It will be frustrating for readers to not have this extra information that can readily be generated. I understand that SMFAs are labor intensive, but the number of experiments that would be needed to address the points above does not need to be extensive. Note that rough estimates of potencies will be acceptable, e.g. understanding whether activity drops below 80% somewhere between xx and yy ug/mL.

The argument that many other papers did not include titrations does in my opinion not hold since Tang et al. included a titration of their most potent mAbs in the supplementary data S7, Miura et al. tested mAbs with no activity (so why titrate?) and the other papers used the mAbs to identify new potent epitopes than presenting the mAbs as transmission blocking tools.

Altogether, I strongly believe that additional titrations are needed and am supported in this by the other reviewers. These extra data, irrespective of the outcome, will certainly strengthen the paper.
Response: We respectfully disagree with Reviewer 2 and based on our previous response to the first revision, we do not believe that titrations are required.

Reviewer #3: Not required.

Part III – Minor Issues: Editorial and Data Presentation Modifications
Please use this section for editorial suggestions as well as relatively minor modifications of existing data that would enhance clarity.

Reviewer #1: (No Response)

Reviewer #2: Most of my minor comments have been addressed, thank you. I would just like to suggest to present the nanobody concentrations in the SMFA figure legends, instead of the absolute amounts. This will help the readers to interpret the data more quickly, rather than having to do the calculations themselves.
Response: As requested, we have modified the text in Figure Legend 2C as follows: "Blood meals included heat-inactivated human serum, 100 µg/mL of nanobody-Fcs or 200 µg/mL TB31F in PBS or 10 µL PBS."

Reviewer #3: No
